# Topochemical control of the photodimerization of aromatic compounds by γ-cyclodextrin thioethers in aqueous solution

**DOI:** 10.3762/bjoc.9.217

**Published:** 2013-09-12

**Authors:** Hai Ming Wang, Gerhard Wenz

**Affiliations:** 1 Organic Macromolecular Chemistry, Saarland University, Campus Saarbrücken C4 2, 66123 Saarbrücken, Germany

**Keywords:** acenaphthylene, anthracene, coumarin, cyclodextrins, photodimerization, quantum yield, stereoselectivity

## Abstract

The formation of soluble 1:2 complexes within hydrophilic γ-cyclodextrin (γ-CD) thioethers allows to perform photodimerizations of aromatic guests under controlled, homogenous reaction conditions. The quantum yields for unsubstituted anthracene, acenaphthylene, and coumarin complexed in these γ-CD thioethers were found to be up to 10 times higher than in the non-complexed state. The configuration of the photoproduct reflected the configuration of the dimeric inclusion complex of the guest. Anti-parallel orientation of acenaphthylene within the CD cavity led to the exclusive formation of the *anti* photo-dimer in quantitative yield. Parallel orientation of coumarin within the complex of a CD thioether led to the formation of the *syn* head-to-head dimer. The degree of complexation of coumarin could be increased by employing the salting out effect.

## Introduction

Photochemical reactions have been considered highly attractive for a long time because they often lead to products that are otherwise virtually inaccessible by thermal reactions. The syntheses of highly strained molecules such as cubane [1] and pagodane [2] are famous examples. However, it is often difficult to predict and control the outcome of photochemical transformations in homogeneous media, and various mixtures of products are obtained. Pre-organization of the reactants in the solid state [3] or by various templates in solution has been the best solution to this problem [4–5].

Cyclic host molecules large enough to accommodate two reacting molecules are the smallest possible templates for the control of photoreactions. Such a host provides a well-defined nano environment, a so-called molecular reaction vessel [6], which can catalyze and direct particular transformations. Calixarenes [7], cucurbiturils [8–9], and cyclodextrins (CDs) [10–16] have already been employed in template controlled photoreactions. Among these hosts CDs offer the advantage of being soluble in water at neutral pH, which is the most favourable solvent for performing photoreactions because of its high transmittance and stability to UV light. Inclusion in CDs not only improves the quantum yields of photodimerizations but also gives rise to high regio- and stereo-selectivities [15–18] However, the application of native CDs for fully hydrophobic reactants is hampered by the fact that their inclusion compounds are nearly insoluble in water leading to undesirable heterogenous reaction conditions [19]. Yields of heterogenous photoreactions generally depend on the particle size of the educt phase because the penetration depth of the incident light is limited.

**Figure 1:**
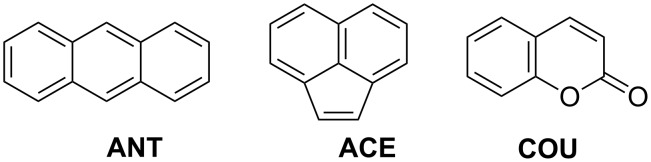
Chemical structures of selected aromatic guests: anthracene, **ANT**; acenaphthylene, **ACE**; and coumarin, **COU**.

**Figure 2:**
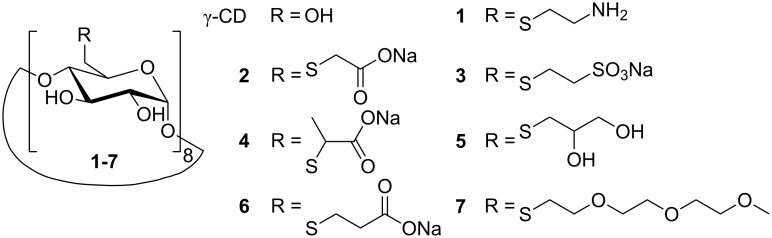
Structures of γ-CD and γ-CD thioethers **1**–**7**.

Recently, we synthesized a series of highly water-soluble per-6-deoxy-thioethers of β- and γ-CD, which are able to solubilize hydrophobic, nearly insoluble guest molecules, such as camptothecin [20], 1,4-dihydroxyanthraquinone [21], betulin [22], benzene and cyclohexane derivatives [23], and even C_60_ [24] in water. Furthermore, their respective γ-CD thioethers form highly water-soluble 1:2 complexes with polycyclic aromatics, such as naphthalene, anthracene (**ANT**), and acenaphthylene (**ACE**) [25]. In the following we report on the photochemistry of **ANT**, **ACE** and coumarin (**COU**), templated by complexation in several hydrophilic γ-CD thioethers **1**–**7** (Figure 1 and Figure 2). Special attention will be paid to both the quantum yields Φ and stereoselectivities of the photodimerizations.

## Results and Discussion

### Photodimerization of anthracene

Due to the fact that the inclusion compounds of **ANT** in native β-CD or γ-CD are completely insoluble in water, only the aqueous photochemistry of hydrophilic **ANT** derivatives, such as anthracene-2-carboxylate (**ANT**-2-COONa) [12], and anthracene-2-sulfonate (**ANT**-2-SO_3_Na) [26], have been investigated so far. The quantum yields of photodimerization increase dramatically from 5 to up to 50% by complexation of these guests with β-CD or γ-CD, as found by Tamaki et al. [26–27]. Formation of 2:2 complexes with β-CD and 1:2 (host/guest) sandwich complexes with γ-CD were made responsible for the observed increase in the quantum yields.

The photodimerization of unmodified **ANT** could be performed for the first time homogenously in aqueous solution, because of the high solubilities of **ANT** complexed by γ-CD thioethers **1**–**7** in water. The highest concentration, [**ANT**] = 0.04 mM, was achieved with a 6 mM solution of host **1** [25]. The respective binding constants *K* [25], and quantum yields Φ for monochromatic irradiation, are listed in Table 1. The values of Φ in the presence of 6.0 mM γ-CD thioethers were very high (16–33%) depending on the side groups of the host. Derivative **4** with thiolactate side groups, performed best. The observed high quantum yields were attributed to the tight sandwich-like packing of the two **ANT** molecules within these hosts, which was already demonstrated previously by fluorescence measurements [25]. The quantum yield Φ did not correlate with the binding constant *K*, possibly because dissolved **ANT** is mainly converted in the complexed state in presence of hosts **1**–**7**. Only a small portion of **ANT** remains in the free state due to its low solubility (0.4 μM, [25]). Instead, packing of the two **ANT** molecules within the complex seems to determine the quantum yield Φ.

**Table 1:** Influence of CDs on the quantum yield Φ of the photodimerization of **ANT** and 1:2 binding constant *K* [25].

Host^a^	Φ^b^ [%]	*K*/10^9^ [M^−2^]

**1**	29	22.8
**2**	16	7.7
**3**	29	6.8
**4**	33	3.4
**5**	25	3.7
**6**	27	6.5
**7**	19	7.7

^a^Concentration of γ-CD thioethers **1**–**7** was 6.0 mM. ^b^For λ = 350 nm, experimental error ± 5 %.

### Stereoselective photodimerization of acenaphthylene

**Scheme 1:**
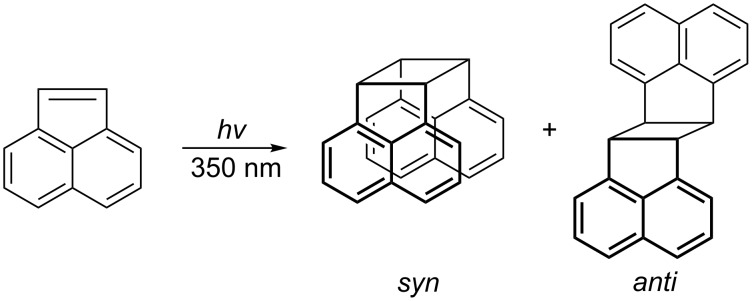
Photodimerization of **ACE**.

The photodimerization of **ACE** generally leads to mixtures of two isomeric cyclobutane derivatives, namely the *syn* and the *anti* dimers, as shown in Scheme 1. The quantum yields for the reactions in both aqueous and organic media are known to be rather low, 1% < Φ < 5%, as summarized in Table 2 [28–29]. Only the addition of solvents with heavy atoms, such as ethyl iodide, leads to satisfactory quantum yields (up to 17%). This increase is accompanied by an increase in the relative amount of the *anti* isomer. The increases of both the quantum yield and amount of the *anti* product – called the heavy atom effect – was attributed to an increased population of triplet states [29–31]. Heavy atoms close to the excited entity accelerate the rate of spin-orbit coupling interactions between states of different spin multiplicities and consequently facilitate intersystem crossing. However, supramolecular control of the packing of **ACE** leads to a significant improvement of the stereoselectivity. **ACE** was complexed in a cavitand nanocapsule [32] and in a Pd nanocage [33] in aqueous solution, both giving rise to the exclusive formation of the *syn* isomer. Unfortunately, the quantum yields have not been reported for these systems so far.

**Table 2:** Quantum yields Φ, distributions of the photodimers, and binding constants *K* [25] of **ACE** for various reaction medi*a*.

Medium	Φ [%]	*syn* [%]	*anti* [%]	*K*/10^6^ M^−2^

Water [32]	–	40	60	–
water/ methanol [28]	3	–	–	–
**3**^a^	28^b^	0^c^	100^c^	37
**6**^a^	12^b^	0^c^	100^c^	27

^a^This work, host concentration 6 mM. ^b^For λ = 300 nm, experimental error ±5%. ^c^Experimental error ±2%.

**Figure 3:**
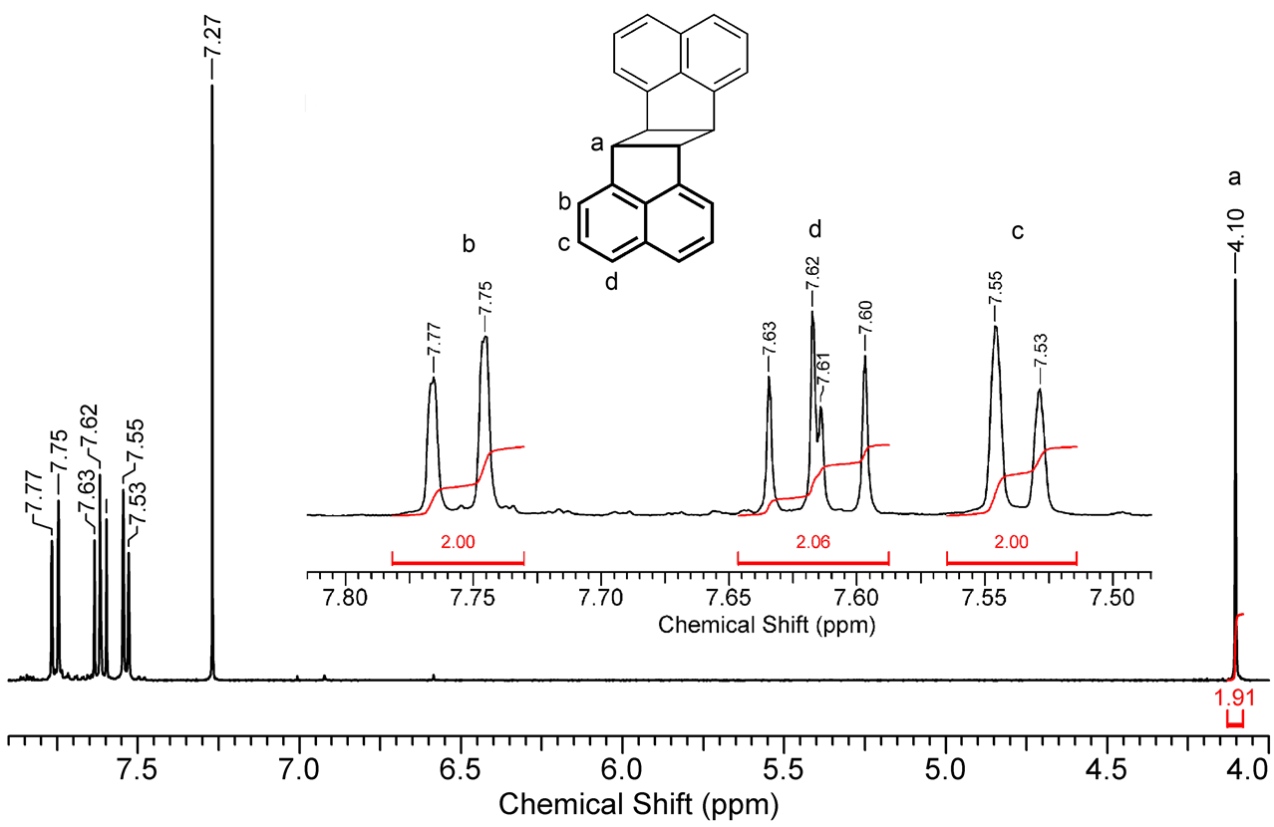
^1^H NMR spectrum of the photo product of **ACE** in the presence of γ-CD thioether **3** in CDCl_3_.

**ACE** also formed inclusion compounds with β-CD thioethers **1**–**7,** which are soluble in aqueous medium. The highest concentrations of **ACE**, [**ACE**] = 1.7 and 1.3 mM, were achieved with 6 mM solutions of hosts **3** and **6**, respectively. The photodimerization of **ACE** complexed in hosts **3** and **6** proceeded under homogenous conditions and furnished nearly quantitatively the *anti* isomer within 12 h. The quantum yields, reaching Φ = 28%, were unprecedentedly high, significantly higher than that of free **ACE** in organic solvents such as toluene (5%), ethanol (4%), and methanol/water (20% v/v, 3%) [28]. The ^1^H NMR signals of cyclobutane (for the *syn* photodimer, δ_H_ = 4.84 ppm; for the *anti* photodimer, δ_H_ = 4.10 ppm) were monitored to analyze the isomeric ratio [34–35]. As shown in Figure 3, only the proton signal at δ_H_ = 4.10 ppm was observed indicating the exclusive formation of the dimer in the *anti* configuration.

**Figure 4:**
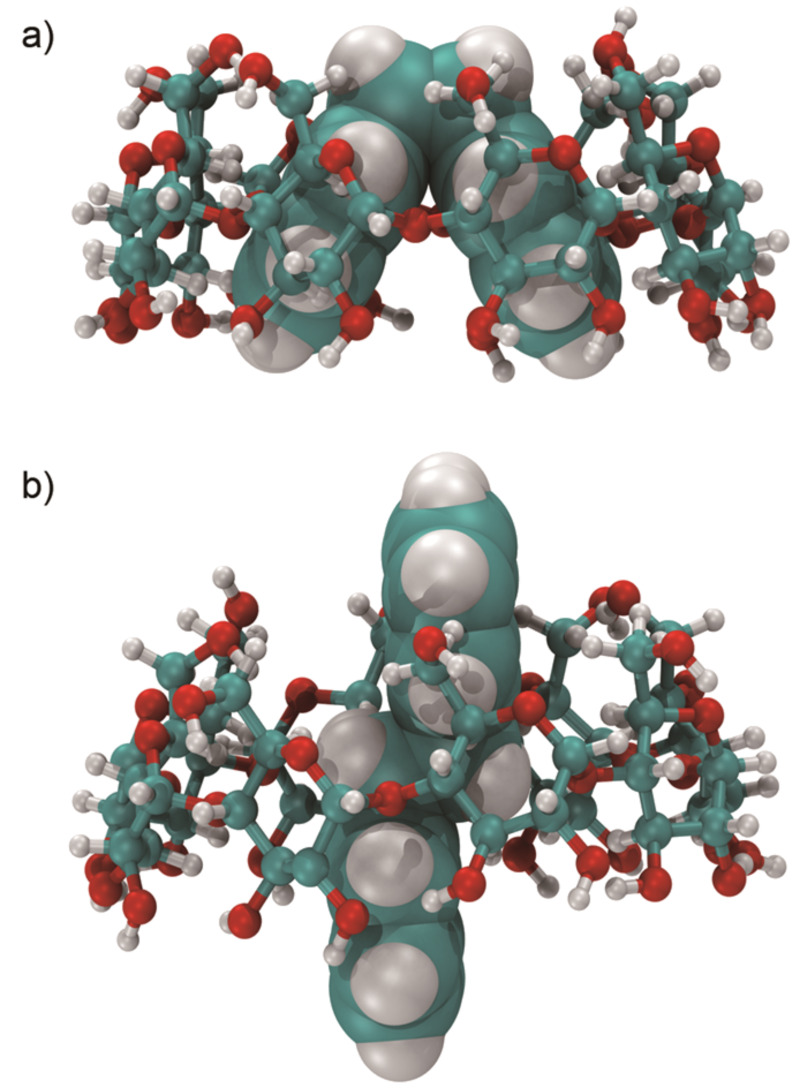
Schematic drawing of the **ACE** photodimers in γ-CD: a) the *syn* photodimer and b) the *anti* photodimer. The rendering was performed with VMD 1.8.7 [37].

Because other hosts favored the *syn* dimer, the total preference of the *anti* dimer was indeed surprising at first, but there are several explanations for the observed *anti* specificity:

(a) **ACE** is tightly surrounded by the seven sulfur atoms of host **3**, which may lead to an increased population of the triplet state and consequently to a heavy atom effect, which favors the formation of the *anti* dimer. Other CD derivatives with heavy atoms attached, e.g., 6-deoxy-iodo-CDs, are known to even enable room temperature phosphorescence of an excited guest [36].

(b) Moreover, according to the results of the quantum mechanical calculations [25,34] the preferential anti-parallel alignment of the **ACE** dimer within the CD cavity also favors the formation of the *anti* dimer.

(c) The *anti* dimer appears to better fit into the γ-CD cavity than the *syn* isomer, as depicted in Figure 4.

### Stereoselective photodimerization of coumarin

**Figure 5:**
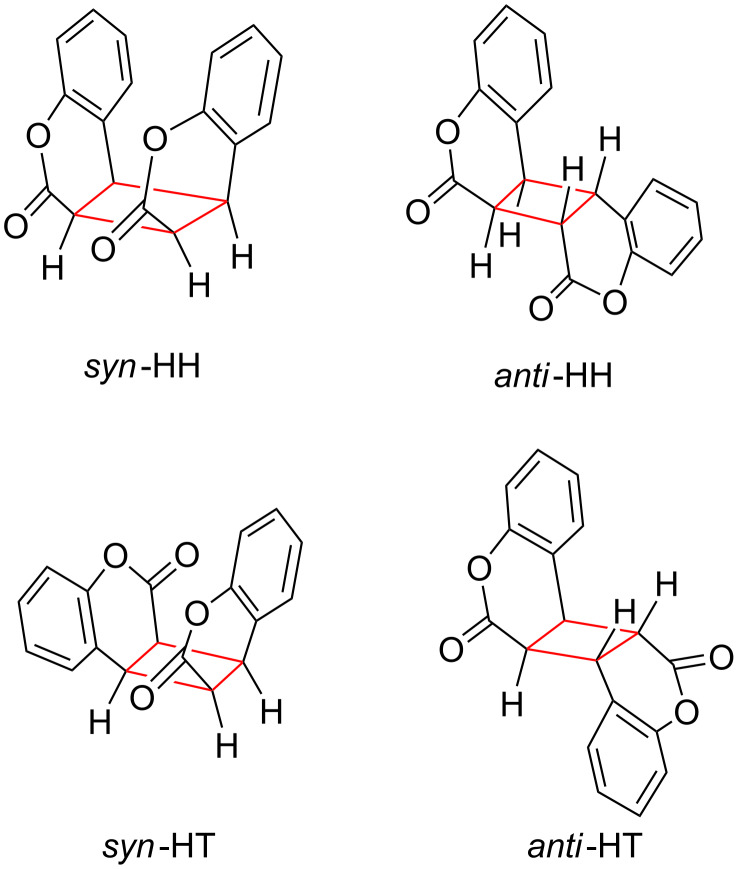
Structures of **COU** photodimers.

The photochemistry of **COU** and its derivatives is rather complex because the quantum yield and distribution of products strongly depend both on the solvent [38] and the concentration of **COU** [39]. In principle, four stereoisomeric dimers are conceivable: *syn* Head-to-Head (*syn*-HH), *anti*-HH, *syn*-Head-to-Tail (*syn*-HT), and *anti*-HH, shown in Figure 5. The photodimerization is very slow in nonpolar media, Φ < 10^−3^% and mainly leading to the *anti*-HH dimer [39]. In contrast, polar protic solvents, like water, increase both the quantum yield and the amount of the *syn*-HH dimer, exemplified in Table 3. The singlet state has a very short lifetime because of rapid intersystem crossing and self-quenching and it reacts to form the *syn*-HH dimer, while the triplet state, with a longer lifetime, furnishes the *anti*-HH isomer [38,40]. Because most often mixtures of *syn* and *anti* isomers were obtained after irradiation of **COU**, any supramolecular control of the dimerization was highly desirable.

**Table 3:** Distribution of the photodimers of **COU** for various media.

Host	Product distribution [%]^a^			

	*syn*-HH	*anti*-HH	*syn*-HT	*syn*/*anti*

water	59	9	32	10
γ-CD^b^	52	17	31	4.7
**1**^b^	58	16	26.5	5.4
**2**^b^	87	5	8.4	19.8
**3**^b^	57	16	26.6	5.1
**4**^b^	71	8	20.9	11.8
**5**^b^	72	9	19.1	10.0
**6**^b^	71	10	18.5	8.6
**7**^b^	51	10	38.6	8.5

^a^Experimental error ± 3%. ^b^6 mM solutions in water.

Photodimerization of a 15 mM aqueous solution of **COU** proceeded with a low quantum yield Φ *=* 0.11% and afforded mainly the *syn*-HH isomer with a reasonable selectivity of *syn*/*anti* = 10, as previously described [38–39]. Photodimerization of 6-methyl-**COU** complexed in cucurbituril CB [8] furnished the *syn*-HT isomer as the main product [41–42]. The analogous effect of β-CD was investigated by Moorthy et al. in 1992. They isolated the *syn*-HH dimer in 64% yield after irradiation for 135 h of the *crystalline* (2:2) inclusion compound of **COU** in β-CD [43]. Therefore, it was very interesting to investigate the supramolecular control exerted by γ-CD thioethers **1**–**7** in aqueous solution. The composition of the mixture of isomeric photodimers was determined from the intensities of the ^1^H NMR signals of the cyclobutane protons, which were assigned according to previous work [40]. The results including the respective *syn*/*anti* ratios are listed in Table 3. In comparison, a 6 mM solution of native γ-CD caused a diminished selectivity of *syn*/*anti* = 4.7.

Surprisingly, the γ-CD thioethers did not behave uniformly. The anionic host **2** increased the selectivity of *syn*/*anti* to 19.8, while the cationic host **1** produced low selectivity, similar to the native γ-CD. The 87% yield of the *syn*-HH dimer obtained with the best host **2** was still not sufficient for any preparative application.

The lower stereoselectivity of the photodimerization of **COU** compared to that of **ACE**, which was achieved with the γ-CD thioethers, was attributed to the much higher aqueous solubility of **COU** (15 mM) compared to the solubility of **ACE** (0.067 mM) [25]. Because the concentration of the free guest [G]_free_ is determined by two coupled thermodynamic equilibria (Equation 1). The 2-phase solubility equilibrium is expressed by Equation 2 and the equilibrium of complexation by the CD host is formulated in Equation 3. The guest dissolves until the concentration of free guest is equal to the solubility of the guest, [G]_0_ = [G]_free_ [44–45]. Consequently, the lower the solubility of the guest in water, the lower the fraction of uncomplexed guests. The lower the concentration of the free guest, the lower the contribution of undesirable and non-specific photodimerization to the total quantum yield, according to Equation 4. Consequently, lowering the aqueous solubility of **COU** by the addition of salt, taking advantage of the so-called “salting-out effect” [46] appeared to be a plausible way to enhance the reaction selectivity of the *syn*-HH dimer.

[1]
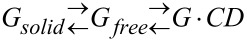


[2]
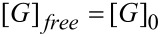


[3]
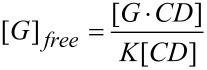


[4]
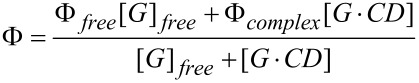


**Figure 6:**
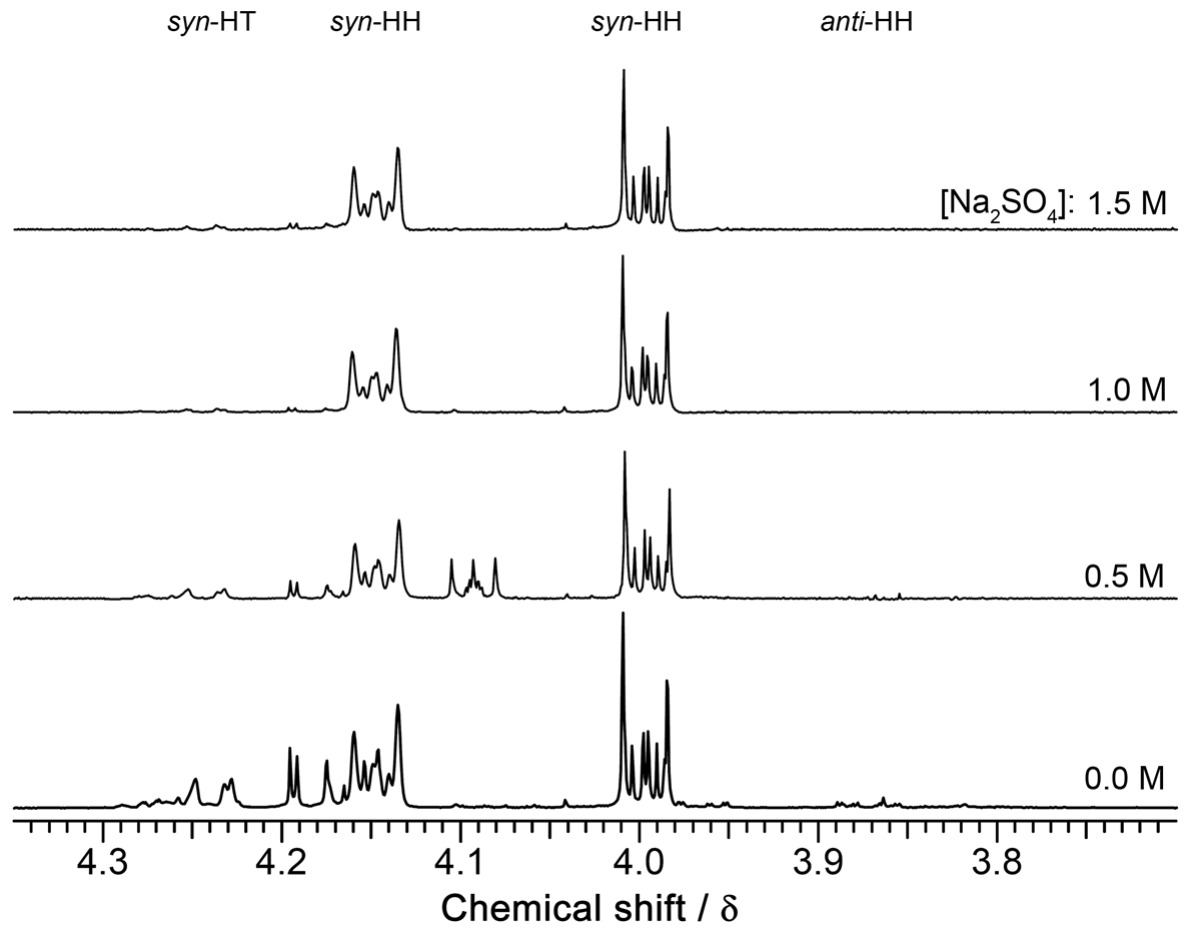
Partial ^1^H NMR of the photodimers formed after irradiation of **COU** at various concentrations of Na_2_SO_4_ in the presence of 6 mM γ-CD thioether **2**.

The ^1^H NMR spectra of the crude photoproducts indeed showed a striking effect from changes in the salt concentration. The signals of the *syn*-HT and *anti*-HH isomers significantly diminished as shown in Figure 6. At a high salt concentration, [Na_2_SO_4_] = 1.5 M, almost pure *syn*-HH dimer was formed exclusively. The compositions, obtained from the NMR intensities, are listed in Table 4. The observed improvement in the quantum yield and stereoselectivity by the addition of salt was quantitatively described taking into account the decrease of the solubility of **COU** by the salting-out effect. As shown in Table 5, the aqueous solubility of **COU** is diminished by nearly a factor of 6 as a result of raising the salt concentration to 1.5 M. In parallel, the quantum yield Φ_free_ of free **COU** is diminished approximately linearly from 2.2% to 1.2%.

**Table 4:** Influence of the sodium sulfate concentration on the quantum yield Φ and distribution of photoproducts of **COU** in a 6.0 mM solution of γ-CD thioether **2**.

[Na_2_SO_4_]	Φ^a^	Product distribution [%]^b^		

M	*%*	*syn*-HH	*anti*-HH	*syn*-HT

0.0	3.8	86	5	9
0.5		89	0	11
1.0		95	0	5
1.5	13.0	97	0	3

^a^For λ = 300 nm, experimental error ±3%. ^b^Experimental error ±2%.

Both effects based on the salt concentration led to a significant reduction in the contribution of the photodimerization of free **COU** to the total quantum yield Φ in Equation 4. Taking into account the molar fraction of the formation of *syn*-HH in water 
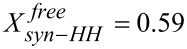
 from Table 3 and in the complex
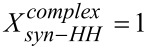
, and assuming the quantum yield for the complex as Φ_complex_ = 19%, the total molar fraction 
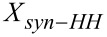
 was calculated according to Equation 5, which was derived from Equation 4. The concentration of complexed **COU**, [**COU**]*_C_*, was determined as the increase in the solubility of **COU** upon the addition of the host. The calculated total quantum yields and molar fractions of the *syn*-HH isomer, listed in Table 5, are in good agreement with the measured values, listed in Table 4. It also shoes, that the binding constant *K*, calculated according to the law of mass action for the 1:2 (host/guest) complex, tremendously increases with the salt concentration leading to a strong increase of the complexed portion of **COU**. In contrast to the previous **ANT** system, the quantum yield of **COU** photodimerization increases with the binding const *K*, because *K* is much lower for **COU** than for **ANT** so that contribution of free **COU** is not negligible.

[5]
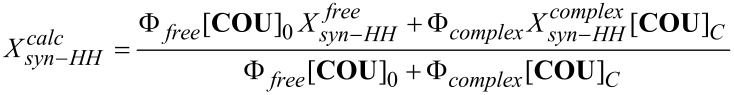


**Table 5:** The quantum yields, calculated according to (4) and the fractions of the *syn*-HH dimer, calculated according to (5), for the photodimerization of **COU** in a 6.0 mM solution of γ-CD thioether **2** and binding constant *K* as a function of salt concentration.

[Na_2_SO_4_] M	[**COU**]_0_ mM	[**COU**]*_C_* mM	Φ*^calc^* %	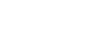 %	*K*/10^3^ M^−2^

0.0	15.2	3.1	5.0	85	2
0.5	8.5	4.8		94	30
1.0	4.7	4.8		97	97
1.5	2.6	5.5	13.2	99	1000

[**COU**]_0_ solubility of **COU**, [**COU**]*_C_* concentration of complexed **COU**, *K* binding constant of **COU** complexed in **2** from solubility measurements.

As a result, the *syn*-HH isomer is exclusively formed from the complex with CD derivative **2,** with a quantum yield approximately ten times higher than that observed without complexation. The tight packing of the two **COU** molecules within the host was made responsible for the significantly increased quantum yield. The heavy atom effect of the S-atoms of CD derivatives **1**–**7** should facilitate intersystem crossing to the triplet state and therefore support the formation of the *anti*-HH dimer. Because this preference does not hold true in this case, especially for host **2**, the multiplicity of the excited state seems not to be the dominant factor, but rather the pre-organization of the guest molecules in the CD cavity prior to excitation has the greatest effect. The distribution of the stereoisomers appears to be mainly topochemically [5] controlled because of the short lifetime of the excited state of **COU**.

Quantum mechanical calculations of the structures and interaction energies Δ*E* of the four **COU** dimers were performed using the Gaussian 03 software package to investigate the favored packing [47]. The aromatic dimers were fully optimized at the MP2/6-31G* level without any symmetry restriction during the computation. The interaction energies Δ*E* of the *syn*-HH, *anti*-HH, *syn*-HT, and *anti*-HT dimers of **COU** were −32.9, −29.3, −24.0, and −22.5 kJ∙mol^−1^, respectively. The Δ*E* value of the **COU** dimer with the *syn*-HH orientation was found to be significantly more negative than that of the dimer with the other orientation. This result can be attributed to relatively efficient π–π stacking [48] The most stable *syn*-HH dimeric aggregate forms the most stable, and therefore, most abundant complex within the *γ*-CD cavity and consequently the main photo-product. Hence, the photodimerization of **COU** within host **2** is a topochemical reaction. This type of topochemical control happening in solution is much more applicable than the classical topochemical control occurring in the crystalline state [3,43,49].

## Conclusion

Aromatic guests **ANT**, **ACE**, and **COU** form 1:2 (host/guest) complexes within γ-CD thioethers **1**–**7**, which photodimerized approximately 10 times faster in aqueous medium than the same uncomplexed guests in aqueous solution. Since these photo dimerizations of hydrophobic guests are performable at neutral pH in optically clear solution in water they are suitable for preparative applications. Isolation of the photoproduct from aqueous solution by liquid/liquid extraction simplifies the scale up. Yields of photodimer based on the complex were nearly quantitative.

The observed supramolecular catalysis was explained by the high pre-organization resulting from the tight packing of the two guests in the cavity, which allows for photoreactions even for short-lived excited states. In comparison to other confined and ordered media, most γ-CD thioether complexes offer the advantages of very high quantum yields and excellent stereochemical control. Because the product formation is mainly topochemically controlled, photoproducts are predictable by quantum mechanical optimization of the corresponding dimeric aggregates in vacuo.

This work also showed that the addition of salt can further improve supramolecular control by suppression of the free photoactive species.

## Experimental

**General**: Guests (**ANT**, **ACE**, and **COU)** and potassium ferrioxalate were purchased from Sigma-Aldrich. Unless otherwise stated, all chemicals were used as received. *γ*-CD was donated by Wacker and dried in vacuo at 100 °C overnight before use. The γ-CD thioethers were synthesized as previously described [25] and purified by nanofiltration (molecular weight cut-off of 1000 Da with demineralized water). Teflon syringe filters (0.22 μm) from Roth (Karlsruhe, Germany) were used to remove insoluble material before taking UV–vis and fluorescence measurements and the photoirradiation experiments. The UV–vis spectra of aqueous samples were taken on a Perkin Elmer Lambda 2 spectrometer (*λ*: 200–600 nm) using quartz cells with a 1 cm or 1 mm optical path at 298 K. The fluorescence spectra were recorded in a JASCO spectrophotometer using quartz cells of 10.0 mm path at 298 K. All of the NMR spectra were recorded on a Bruker 400 MHz NMR spectrometer at 298 K using the solvent peaks as internal references, and the coupling constants (*J*) were measured in Hz.

**Photoreaction procedures**: Aqueous solutions of the native CDs or *γ*-CD thioethers (25 mL, 6.0 mM) were added into glass vials containing excess amounts of the guest molecules. To evaluate the salting-out effect on the photo-product distribution of **COU**, different amounts of sodium sulfate were added to the vials to obtain the desired final salt concentration (Na_2_SO_4_ concentration: 0, 0.5, 1.0, and 1.5 M). Sample solutions were thoroughly degassed using sonication. The vials were tightly sealed, protected from light, and magnetically stirred at room temperature. After 72 h, the resulting suspensions were filtered through syringe filters. The concentrations of the reactants in the CD solutions were determined from the UV–vis extinction coefficients at the absorption maxima.

The aqueous solutions of the inclusion complexes were transferred into 50 mL quartz round-bottomed flasks and bubbled with nitrogen gas for 20 min with stirring before photo-irradiation. Photodimerization of the samples was carried out using a medium-pressure mercury lamp (166.5 W, Heraeus Noblelight GmbH, UVB) as a light source. After irradiation for 12 h at room temperature under a nitrogen atmosphere, the photoproducts formed were extracted with chloroform and then analyzed by ^1^H NMR [34]. No starting material was found anymore after this time of reaction. It is worth mentioning that a precipitate was formed after irradiation of the aqueous solution of the inclusion complex of **ACE** with γ-CD thioether.

**Molecular modeling**: A previously described procedure was used [25]. The geometries of the orientational isomers of the **COU** gas-phase dimer were fully optimized without any symmetry restriction at the MP2/6-31G* level of theory by using the Gaussian 03 (E.01) software package [47]. Corrected interaction energies (∆*E*) corresponding to the optimized aromatic dimers were evaluated using the equation ∆*E* = *E*_dimer_ – 2*E*_monomer_ at the MP2/6-31G* level, where *E*_dimer_ and *E*_monomer_ are the energies of the aromatic dimer and the monomer, respectively [50].

## Supporting Information

File 1 Quantum yield measurement.
