# Supplementary material for: Topochemical control of the photodimerization of aromatic compounds by γ-cyclodextrin thioethers in aqueous solution
Source: Beilstein J Org Chem. 2013 Sep 12;9:1858–66. doi: 10.3762/bjoc.9.217 (PMC3778407; doi:10.3762/bjoc.9.217)
Supplement: File 1 — Quantum yield measurement. [file Beilstein_J_Org_Chem-09-1858-s001.pdf]

## Supporting Information

for

# Topochemical control of the photodimerization of aromatic compounds by γ-cyclodextrin thioethers in aqueous solution

Hai Ming Wang, Gerhard Wenz*

Address: Organic Macromolecular Chemistry, Saarland University, Campus Saarbrücken C4 2, 66123 Saarbrücken, Germany.

Email: Gerhard Wenz - g.wenz@mx.uni-saarland.de

* Corresponding author

**Quantum yield measurement**: the definition of a quantum yield (Φ) for a photochemical reaction at a given irradiation wavelength (λ) was determined according to equation (1), where $n_r$ is the number of molecules that reacted and $n_p$ is the number of photons at the irradiation wavelength λ that were absorbed by the reactant [1-4].

$$\Phi = n_r / n_p \qquad (1)$$

$n_r$ was determined as follows: Exactly 3.00 mL of the aqueous solution of host–guest complex (fixed concentration of γ-CD thioether 6 mM) was placed into a quartz cuvette ($d$ = 1 cm) and exposed to monochromatic UV light for a certain period of time. The irradiation wavelengths used for **ANT**, **ACE**, and **COU** were 350, 300, and 300 nm, respectively. The amount of reacted guest was determined from the decay of its UV extinction using the reported molar peak extinction coefficient (ε of **ANT**, **ACE**, and **COU** 7350, 9090, and 5650 $Lmol^{-1}cm^{-1}$, respectively) [5].

The value for $n_p$ was determined as follows: a 6 mM actinometer (potassium ferrioxalate) solution was prepared by dissolving 2.95 g of potassium ferrioxalate in ca. 800 mL of water. Sulfuric acid (100 mL of 0.5 M) was then added, and the solution was diluted to 1 L and mixed. A quartz cuvette ($d$ = 1 cm) containing 3.00 mL of the actinometer was irradiated under stirring simultaneously during the determination of the quantum yields of the dimerization reactions. Under these conditions potassium ferrioxalate absorbs <99% the light for wavelengths up to 390 nm.

The amount of $Fe^{2+}$ formed was determined by titration with phenanthroline after photoirradiation. An aliquot (0.2 mL) of the actinometer solution was pipetted into a 10 mL calibrated flask. Next, 0.1 mL of buffer (obtained from 600 mL of 1 M sodium acetate and 360 mL of 0.5 M sulfuric acid diluted to 1 L) and 0.2 mL of phenanthroline solution (0.1% w/v) were added into the flask and filled up with water to 10.00 mL. The absorbance of the resulting solution and the unexposed actinometer solution at 510 nm was measured. The amount of ferrous iron was calculated from the absorbance difference using an extinction coefficient of

11,050 L·mol$^{-1}$·cm$^{-1}$.The quantity of ferrous iron was converted to a radiation dose using the reported quantum yield of potassium ferrioxalate at the given wavelength (e.g. $\Phi$ = 1.24 at 313 nm) [4].

## References


1. Tonne, J.; Prinzbach, H.; Michl, J. *Photochem. Photobiol. Sci.* **2002,** *1,* 105-110.
2. Sun, L. Z.; Bolton, J. R. *J. Phys. Chem.* **1996,** *100,* 4127-4134.
3. Harpp, D. N.; Heitner, C. *J. Am. Chem. Soc.* **1972,** *94,* 8179-8184.
4. Hatchard, C. G.; Parker, C. A. *Proc. R. Soc. Lond. A* **1956,** *235,* 518-536.
5. Wang, H. M.; Wenz, G. *Chemistry – An Asian Journal* **2011***,* 2390-2399.
